# Comment on Dhiman, R. et al. Correlation of Non-Polio Acute Flaccid Paralysis Rate with Pulse Polio Frequency in India. *Int. J. Environ. Res. Public Health* 2018, *15*, 1755

**DOI:** 10.3390/ijerph16010013

**Published:** 2018-12-21

**Authors:** Siddalingaiah H.S.

**Affiliations:** Department of Community Medicine, Shridevi Institute of Medical Sciences and Research Hospital, Tumkur 572106, Karnataka, India; hssling@yahoo.com; Tel.: +91-897-196-1783

The authors of the published article ‘Correlation between Non-Polio Acute Flaccid Paralysis Rates with Pulse Polio Frequency in India’ [1] have not considered several important variables in their conclusions of their findings, which might have affected the validity of the correlations.

I would like to highlight several important points:

1. The Acute Flaccid Paralysis (AFP) surveillance data that the authors have used include reported cases of the 0–15 year age group and the rates are calculated forthwith. However, the use of Oral Polio Vaccine (OPV) in Intensified Pulse Polio Immunization (IPPI) or Pulse Polio campaigns is targeted toward children in the age range of 0–5 years. Hence, correlating the two does not actually answer the hypothesis unless some analytical evidence is provided to show that the AFP rate in the 5–15 year age group is not influencing the results.
2. There is the possibility that the sensitivity of surveillance and, therefore, the number of AFP cases reported depended not only on the broadened case definition, to include even atypical or non-classical AFP cases, but also on the focus and efforts in terms of active case search (ACS) visits by the surveillance officers. It is a reality that due to the transitioning plans, a shifting of focus from polio, and the reduced presence of specialized surveillance system workforce from partner agencies such as WHO, especially in Uttar Pradesh and Bihar, the number of ACS visits to the reporting and informer units of the AFP reporting network have decreased considerably. This might also be correlated with the non-polio AFP rates in recent years.
3. When comparing the data from states such as Delhi, Karnataka and Kerala, as per the data sheet provided by the authors, one fails to see the correlation observed by the authors for these states. Even the southern and northern parts of states such as Karnataka, with the same frequency of pulse polio campaigns, have widely variable non-polio AFP rates.
4. The frequency of administration of OPV has increased due to routine immunization strengthening efforts, especially in northern states such as Uttar Pradesh and Bihar, stemming from campaigns such as special immunization weeks, Mission Indradhanush, and Gram Swaraj Abhiyan, which have been active from 2013/2014 onwards. The routine immunization OPV doses (5) are reaching a greater percentage of each birth cohort (about 30-40% more in northern states since 2013/2014), which is not accounted for by the authors.
5. There was a change in OPV type during the global switch from tOPV to bOPV in 2016. IPV was introduced prior to the switch to avoid the risk of VDPV emergence. In addition, the complex effects of IPV generated immunity against polio paralysis of any type (WPV, VDPV, or VAPP), when administered simultaneously with bOPV, due to variation in the speed and magnitude of serosal immunity generation by IPV vis-à-vis that by bOPV, and the subsequent reduction in the risk of generation of paralytic illness is also not discussed or accounted for. Moreover, the low coverage of IPV, due to global shortages in supply chains, is not accounted for.
6. The possibility of post vaccination paralysis, though described in the literature to be about one in two to three million doses, might apply only to those children who take vaccines for the first time, those who are totally naïve with no pre-existing immunity. This might be much more uncommon in already vaccinated children. As the naïve cohort will remain similar (or with only a slight change caused by the birth rate) irrespective of the number of polio campaign rounds or the routine immunization schedule, the possibility of the OPV induced paralysis rate changing much each year is questionable. Hence, there appears to be no biological plausibility for the conclusions on correlation described by the authors, which should be regarded as spurious unless proved otherwise by further studies.
7. Another important factor, i.e., non-polio enteroviruses (NPEV) causing polio like paralysis, and variations in the temporal and spatial distribution of NPEVs across Indian states are unaccounted for.
8. The authors do not describe how the proportions of actual diagnoses of AFP cases have varied over time, nor what percentage fit into the classical AFP criteria as practiced in the western countries to which the authors have drawn the non-polio AFP rate comparisons of India.

With all of these questions at bay, the findings and inferences described in the article are questionable and should be considered spurious unless proved otherwise by further study and analysis using more rigorous methods.
